# Pictorial review: a step-by-step guide to coronary CT angiography with photon-counting detector CT

**DOI:** 10.1093/bjr/tqag095

**Published:** 2026-04-26

**Authors:** Cheng Xie, Nicholas Clarke, Rafail Kotronias, Kenneth Chan, Leonardo Portolan, Sheena Thomas, Jonathan Denton, Robyn Farrall, Caroline Taylor, Chloe Yeabin Jung, Thomas Halborg, Chrysovalantou Nikolaidou, Andrew Kelion, Nikant Sabharwal, David Holdsworth, Betty Raman, Stefan Neubauer, Charalambos Antoniades

**Affiliations:** Division of Cardiovascular Medicine, Radcliffe Department of Medicine, University of Oxford, West Wing Level 6, John Radcliffe Hospital, Oxford OX3 9DU, United Kingdom; Acute Multidisciplinary Imaging & Interventional Centre, Radcliffe Department of Medicine, University of Oxford, Level 2 John Radcliffe Hospital, Oxford OX3 9DU, United Kingdom; Division of Cardiovascular Medicine, Radcliffe Department of Medicine, University of Oxford, West Wing Level 6, John Radcliffe Hospital, Oxford OX3 9DU, United Kingdom; Acute Multidisciplinary Imaging & Interventional Centre, Radcliffe Department of Medicine, University of Oxford, Level 2 John Radcliffe Hospital, Oxford OX3 9DU, United Kingdom; Division of Cardiovascular Medicine, Radcliffe Department of Medicine, University of Oxford, West Wing Level 6, John Radcliffe Hospital, Oxford OX3 9DU, United Kingdom; Acute Multidisciplinary Imaging & Interventional Centre, Radcliffe Department of Medicine, University of Oxford, Level 2 John Radcliffe Hospital, Oxford OX3 9DU, United Kingdom; Division of Cardiovascular Medicine, Radcliffe Department of Medicine, University of Oxford, West Wing Level 6, John Radcliffe Hospital, Oxford OX3 9DU, United Kingdom; Acute Multidisciplinary Imaging & Interventional Centre, Radcliffe Department of Medicine, University of Oxford, Level 2 John Radcliffe Hospital, Oxford OX3 9DU, United Kingdom; Acute Multidisciplinary Imaging & Interventional Centre, Radcliffe Department of Medicine, University of Oxford, Level 2 John Radcliffe Hospital, Oxford OX3 9DU, United Kingdom; Division of Cardiovascular Medicine, Radcliffe Department of Medicine, University of Oxford, West Wing Level 6, John Radcliffe Hospital, Oxford OX3 9DU, United Kingdom; Acute Multidisciplinary Imaging & Interventional Centre, Radcliffe Department of Medicine, University of Oxford, Level 2 John Radcliffe Hospital, Oxford OX3 9DU, United Kingdom; Division of Cardiovascular Medicine, Radcliffe Department of Medicine, University of Oxford, West Wing Level 6, John Radcliffe Hospital, Oxford OX3 9DU, United Kingdom; Acute Multidisciplinary Imaging & Interventional Centre, Radcliffe Department of Medicine, University of Oxford, Level 2 John Radcliffe Hospital, Oxford OX3 9DU, United Kingdom; Division of Cardiovascular Medicine, Radcliffe Department of Medicine, University of Oxford, West Wing Level 6, John Radcliffe Hospital, Oxford OX3 9DU, United Kingdom; Acute Multidisciplinary Imaging & Interventional Centre, Radcliffe Department of Medicine, University of Oxford, Level 2 John Radcliffe Hospital, Oxford OX3 9DU, United Kingdom; Division of Cardiovascular Medicine, Radcliffe Department of Medicine, University of Oxford, West Wing Level 6, John Radcliffe Hospital, Oxford OX3 9DU, United Kingdom; Acute Multidisciplinary Imaging & Interventional Centre, Radcliffe Department of Medicine, University of Oxford, Level 2 John Radcliffe Hospital, Oxford OX3 9DU, United Kingdom; Acute Multidisciplinary Imaging & Interventional Centre, Radcliffe Department of Medicine, University of Oxford, Level 2 John Radcliffe Hospital, Oxford OX3 9DU, United Kingdom; Division of Cardiovascular Medicine, Radcliffe Department of Medicine, University of Oxford, West Wing Level 6, John Radcliffe Hospital, Oxford OX3 9DU, United Kingdom; Acute Multidisciplinary Imaging & Interventional Centre, Radcliffe Department of Medicine, University of Oxford, Level 2 John Radcliffe Hospital, Oxford OX3 9DU, United Kingdom; Acute Multidisciplinary Imaging & Interventional Centre, Radcliffe Department of Medicine, University of Oxford, Level 2 John Radcliffe Hospital, Oxford OX3 9DU, United Kingdom; Acute Multidisciplinary Imaging & Interventional Centre, Radcliffe Department of Medicine, University of Oxford, Level 2 John Radcliffe Hospital, Oxford OX3 9DU, United Kingdom; Acute Multidisciplinary Imaging & Interventional Centre, Radcliffe Department of Medicine, University of Oxford, Level 2 John Radcliffe Hospital, Oxford OX3 9DU, United Kingdom; Acute Multidisciplinary Imaging & Interventional Centre, Radcliffe Department of Medicine, University of Oxford, Level 2 John Radcliffe Hospital, Oxford OX3 9DU, United Kingdom; Division of Cardiovascular Medicine, Radcliffe Department of Medicine, University of Oxford, West Wing Level 6, John Radcliffe Hospital, Oxford OX3 9DU, United Kingdom; Division of Cardiovascular Medicine, Radcliffe Department of Medicine, University of Oxford, West Wing Level 6, John Radcliffe Hospital, Oxford OX3 9DU, United Kingdom; Division of Cardiovascular Medicine, Radcliffe Department of Medicine, University of Oxford, West Wing Level 6, John Radcliffe Hospital, Oxford OX3 9DU, United Kingdom; Acute Multidisciplinary Imaging & Interventional Centre, Radcliffe Department of Medicine, University of Oxford, Level 2 John Radcliffe Hospital, Oxford OX3 9DU, United Kingdom

**Keywords:** photon counting, cardiac CT, coronary artery disease

## Abstract

Recent advances in CT technology have led to the advent of the photon-counting detector CT (PCD-CT). The technology has shown significant improvements in CT image quality with high spatial resolution, more accurate differentiation of coronary plaque components, and radiation reductions compared to the energy-integrating detector-CT (EID-CT). However, limited experience exists, and little has been published on the decision-making process for PCD-CT coronary scanning protocols and parameters in clinical practice. In this pictorial review, we showcase our experience in the use of PCD-CT coronary CT angiogram through a step-by-step guide in the form of a decision tree, a series of clinical cases, and a detailed discussion of each scanning protocol to aid the operator in making decisions in commonly encountered clinical scenarios. The guide should also help the operator in setting up a PCD-CT cardiac imaging service.

## Introduction

Coronary CT angiography (CCTA) is widely recognized as the first line noninvasive imaging modality for the investigation of coronary artery disease (CAD).^1–3^ Advances in CT technology have led to the advent of the photon-counting detector CT (PCD-CT). With its efficient and smaller detector pixel size, PCD-CT overcomes many of the technical limitations of traditional energy-integrating detector CT (EID-CT).^4–6^ PCD-CT offers significant improvements in CT image quality with higher spatial resolution, more accurate differentiation of coronary plaque components, and reduced blooming artifacts from calcification and stents.^4–7^ PCD-CT also has advanced spectral imaging capabilities through its ability to categorize incoming photons into discrete energy-level bins.^8^ There is little published advice regarding the decision-making process for selecting the optimal PCD-CT coronary scanning protocol and parameters for individual patients. We showcase our experience in the clinical use of PCD-CT CCTA through a step-by-step guide and a series of clinical cases.

## Coronary CTA acquisition protocol

Coronary CT angiographys are electrocardiographically (ECG) gated and performed on a NAEOTOM Alpha PCD-CT scanner (Siemens Healthcare GmbH, Erlangen, Germany, VA50SP1). Heart rate is lowered if required with intravenous beta-blockers (metoprolol) to target a heart rate of less than 65 beats/min (bpm), and sublingual glyceryl trinitrate spray up to 800 mg is administered according to guidelines.^9^ Using a dual-syringe injector, 80-100 mL of iodinated contrast medium (GE Healthcare OMNIPAQUE350) is administered at an injection rate of 4.5 mL/s, followed by 50 mL flush of normal saline at injection rate of 4.5 mL/s. In selected cases spectral PCD-CT may allow for lower contrast doses to be used without compromising image quality, utilizing monoenergetic images at a lower kiloelectronvolt (keV) to increase vascular attenuation.^10–12^

**Figure 1:**
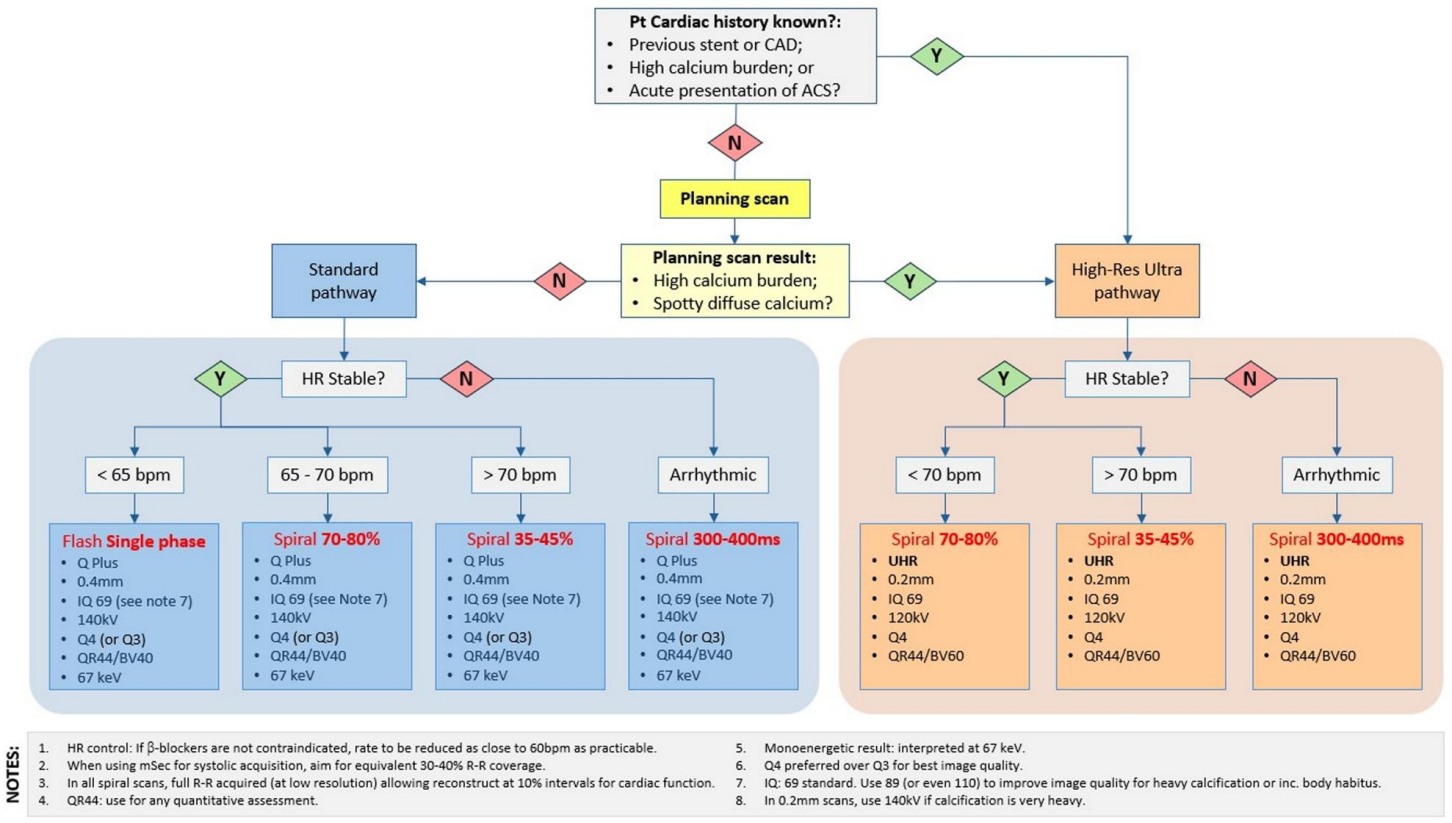
The photon-counting detector CT coronary CTA acquisition protocol decision tree.

Our PCD-CT CCTA acquisition protocol consists of 2 pathways—a standard and an ultra-high resolution (UHR) pathway (Figure 1). There is a tradeoff between image quality and radiation exposure for the UHR pathway. Therefore, the UHR pathway is reserved for patients already known to have CAD (confirmed acute coronary syndrome or stent insertion) or those with coronary calcification identified on a non-contrast planning scan. A non-contrast planning scan is performed on all patients to guide this decision: an ECG-gated FLASH non-contrast acquisition of 144 × 0.4 mm, pitch 3.2, gantry rotation time 0.25 s, 90 kVp, IQ level 6. The image quality (IQ) level has an impact on the quality reference tube current (mAs), hence it is kept low to minimize the radiation dose from the planning scan. The mean and SD of the dose–length product (DLP) of the planning scans are 12.8 ± 5.4 mGy.cm (0.35 mSv^13^) from 40 patients (mean height 172 cm, weight 84 kg).

The planning scan offers a quick qualitative calcification assessment. If the operator finds any calcification in the left main, proximal vessels, or moderate to severe calcification elsewhere, then the UHR protocol is selected. Otherwise, the standard pathway is chosen. Subsequent refinement of the protocol is based on heart rate and rhythm. The heart rate is considered stable if the patient is in sinus rhythm without frequent ectopy or arrhythmia.

## FLASH protocol

**Figure 2:**
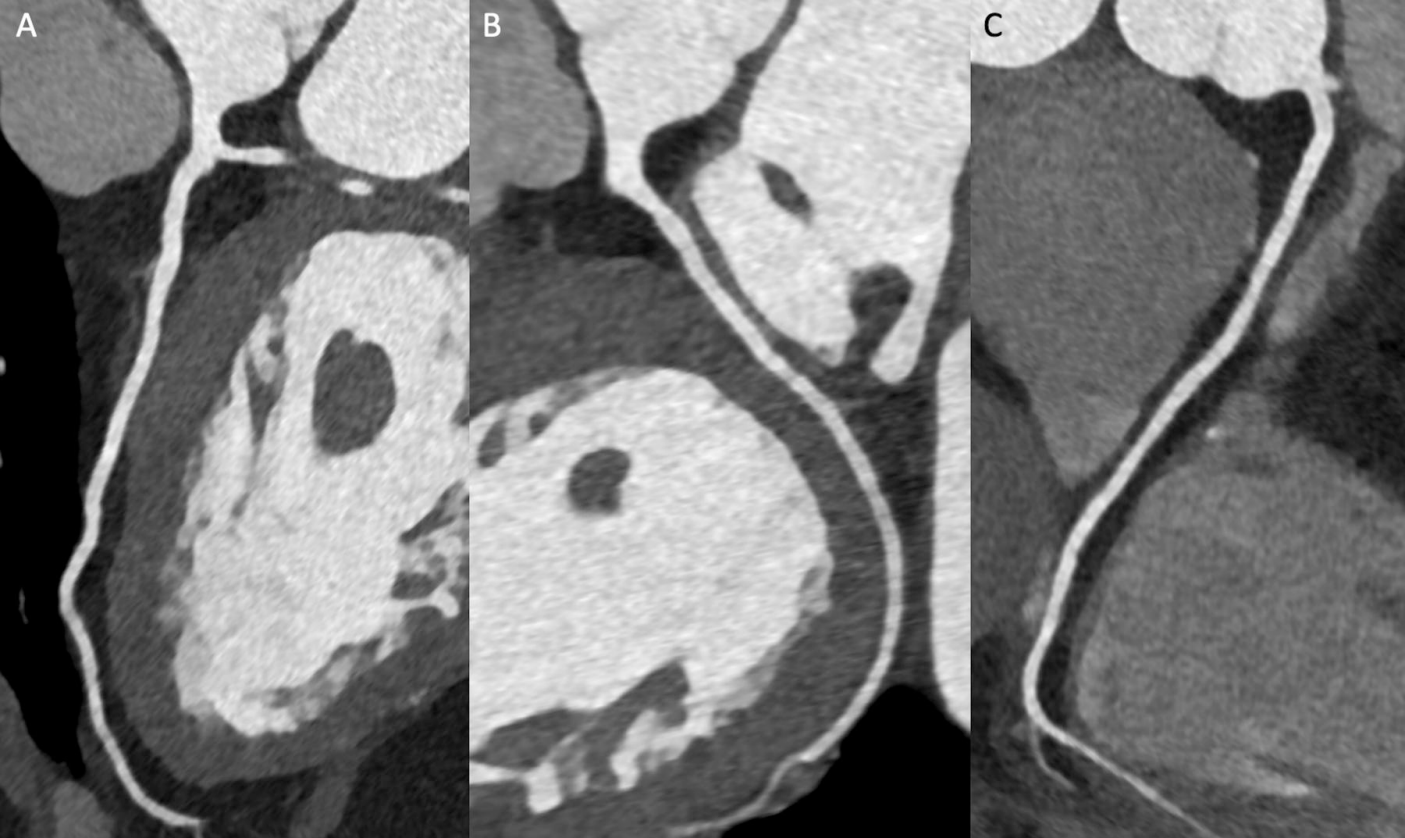
Coronary CTA with the FLASH protocol. Single best-diastolic-phase acquisition with high quality images of the LAD (A), LCX (B) and RCA (C). This is the preferred scanning option for a patient in sinus rhythm and heart rate <65 bpm without coronary calcification on the planning scan.

The FLASH protocol is a single diastolic phase high-pitch helical acquisition (Figure 2) and it is the preferred scanning option due to its low radiation dose. In 623 patients (mean height 173 cm, weight 86 kg) who underwent CCTA according to this protocol had mean and SD dose-length-product (DLP) of 122 ± 69 mGy.cm (3.4 mSv).

**Figure 3:**
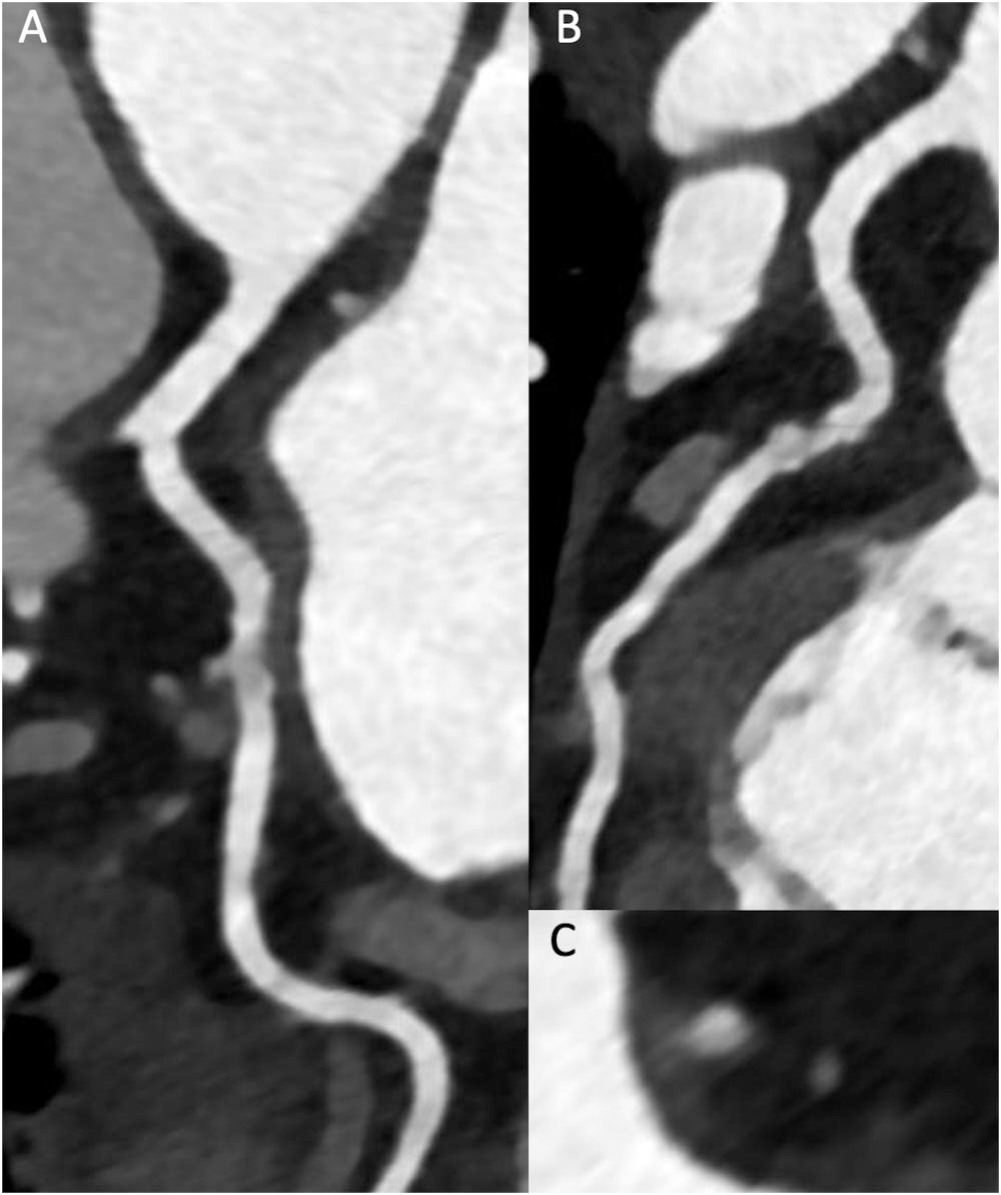
The limitations of the FLASH protocol. The panels (A-C) show reduced luminal contrast due to possible mixed plaque at the proximal LCx. A horizontal motion artifact across in the segment of interest prevented detailed assessment. A moderate luminal stenosis seen on CT was actually severe on the invasive angiogram.

**Figure 4:**
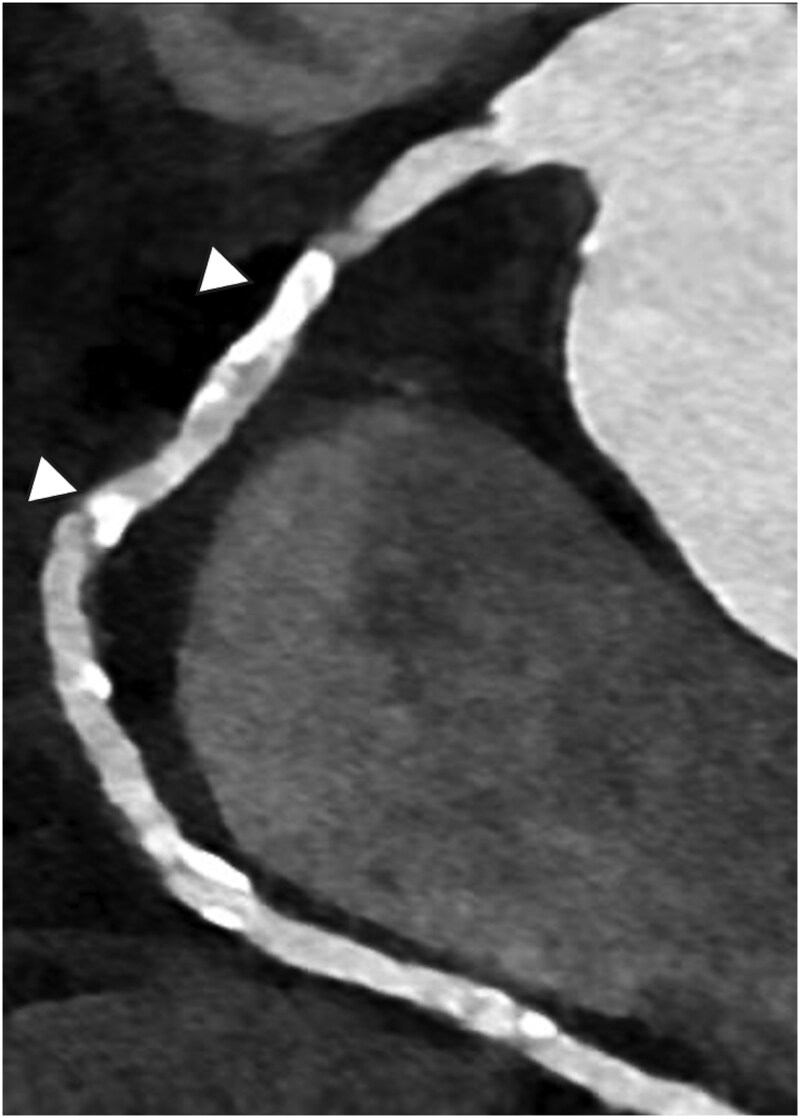
The limitations of the FLASH protocol with severe coronary calcification. A severe luminal stenosis (arrowhead) on CT was actually mild/moderate on invasive angiogram.

This protocol should be selected for patients without known coronary disease who do not have significant calcification on the planning scan and are in sinus rhythm with a heart rate <65 bpm. When the heart rate is ≥65 bpm or there are frequent ectopic beats or arrhythmias, then using the FLASH protocol increases the risk of motion artifacts on the resultant images. Figure 3 illustrates this issue in a case where the proximal circumflex artery (LCx) demonstrated reduced luminal contrast and possibility of a mixed plaque in the same location as a horizontal motion artifact. The single acquired image using the FLASH mode prevented further detailed assessment, and the luminal stenosis was initially graded as moderate on CCTA but was shown to be severe on invasive coronary angiography. Hence, FLASH mode is not recommended in cases with tachycardia or arrhythmia where motion artifacts are more likely to be a problem. Figure 4 demonstrates how heavy coronary calcification can result in blooming artifacts on a FLASH scan that limits accurate luminal assessment; therefore, the UHR scan protocol is the preferred option in these patients.

## Ultra-high resolution (UHR) protocol

**Figure 5:**
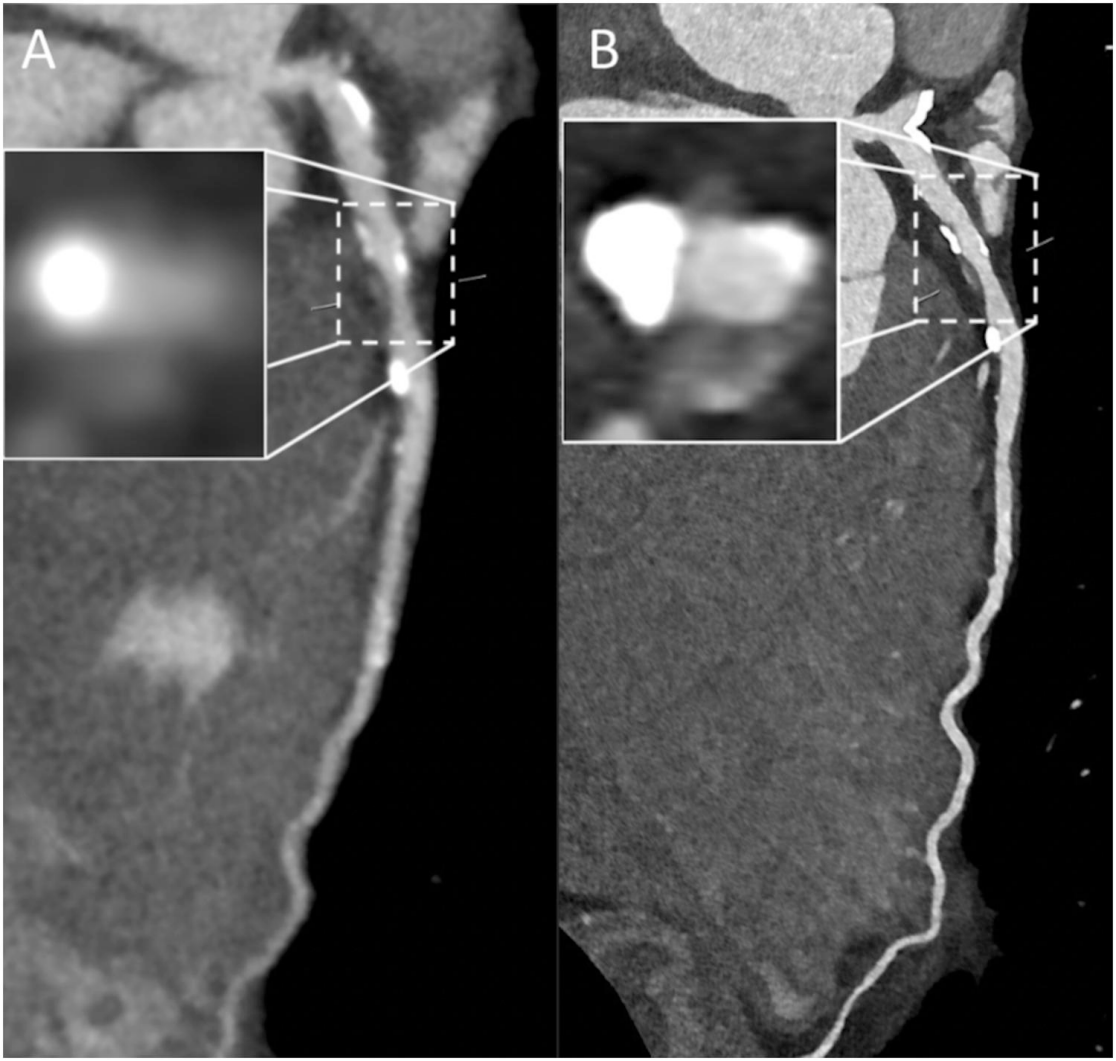
The same calcified plaque reconstructed at 0.6 mm (Panel A) and 0.2 mm (Panel B) slice thickness to demonstrate UHR plaque and luminal assessment to downgrade stenosis severity from severe to mild and prevent unnecessary invasive angiography.

The UHR protocol is obtained with a spiral acquisition and is the protocol of choice when there is a high burden of coronary calcification, complex mixed plaque, and/or a coronary stent. We also recommend it for symptomatic patients with Acute Coronary Syndrome (ACS) where the likelihood of complex vulnerable plaque is high. UHR CCTA utilizes a 0.2-mm slice thickness to provide high spatial resolution, minimal blooming effect from calcification, and accurate differentiation of plaque components. In Figure 5, the same calcified plaque reconstructed at 0.6-mm slice thickness (Figure 5A) and 0.2-mm slice thickness (Figure 5B) illustrates how the improved image quality of UHR mode can downgrade stenosis severity from severe to mild, and this has been shown to lead to reductions in unnecessary invasive angiography.^7^^,^^14^ However, this comes with a radiation dose penalty compared to the standard pathway: in 233 patients (mean height 171 cm, weight 87 kg) who underwent UHR CCTA had mean and SD DLP of 573 ± 254 mGy.cm (16 mSv). Despite this, the radiation dose of UHR scans still remains comparable to or less than that of EID-CT.^15^

**Figure 6:**
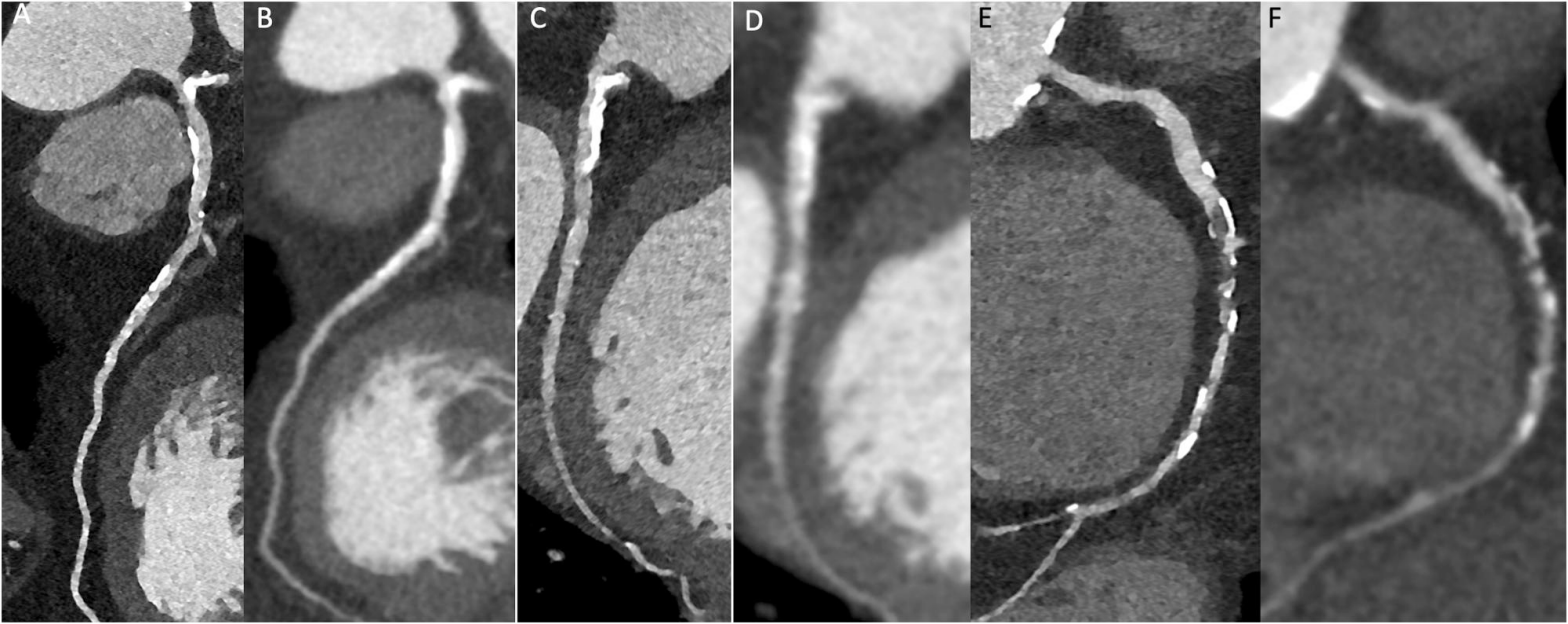
PCD-CT (Panels A, C, and E) vs EID coronary CTA (Panels B, D, and F) in the same patient 1 week earlier. Note the higher spatial resolution and superior plaque differentiation on PCD-CT compared to EID-CT.

**Figure 7:**
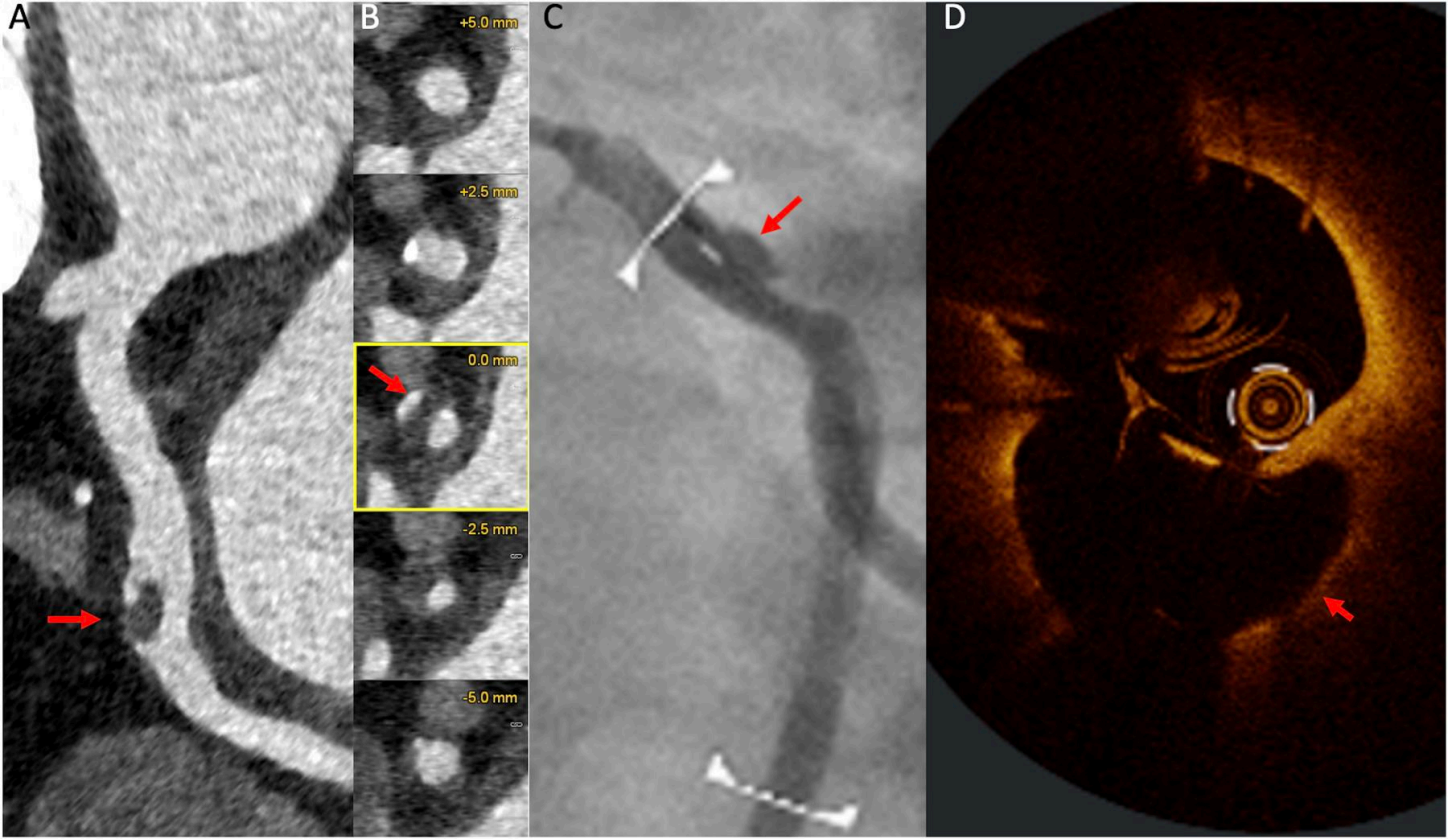
Healed ruptured plaque. UHR images (Panels A and B) show a mixed and complex plaque with a large non-calcified component and a separate cavity (red arrow). This was confirmed on the invasive angiogram (Panel C) showing retrograde filling of the cavity (red arrow), and by direct visualization of the cavity on optical coherence tomography (Panel D).

The case in Figure 6 demonstrates the higher spatial resolution and plaque differentiation on PCD-CT using UHR mode compared to a study performed on the same patient using EID-CT 1 week earlier. The PCD-CT study allows greater confidence in plaque and vessel luminal assessment and can also demonstrate subtle features of plaque rupture. At first sight the case shown in Figure 7 could be interpreted as showing non-calcified plaque causing moderate stenosis. Subsequent detailed assessment on the axial slices (Figure 7B) revealed a mixed complex plaque with a large non-calcified component, and most importantly a separate cavity, which raised the suspicion of a healing ruptured plaque. This diagnosis was confirmed at invasive angiography, which show retrograde filling of the cavity, and by direct visualization of the cavity using optical coherence tomography (Figure 7C and D).

**Figure 8:**
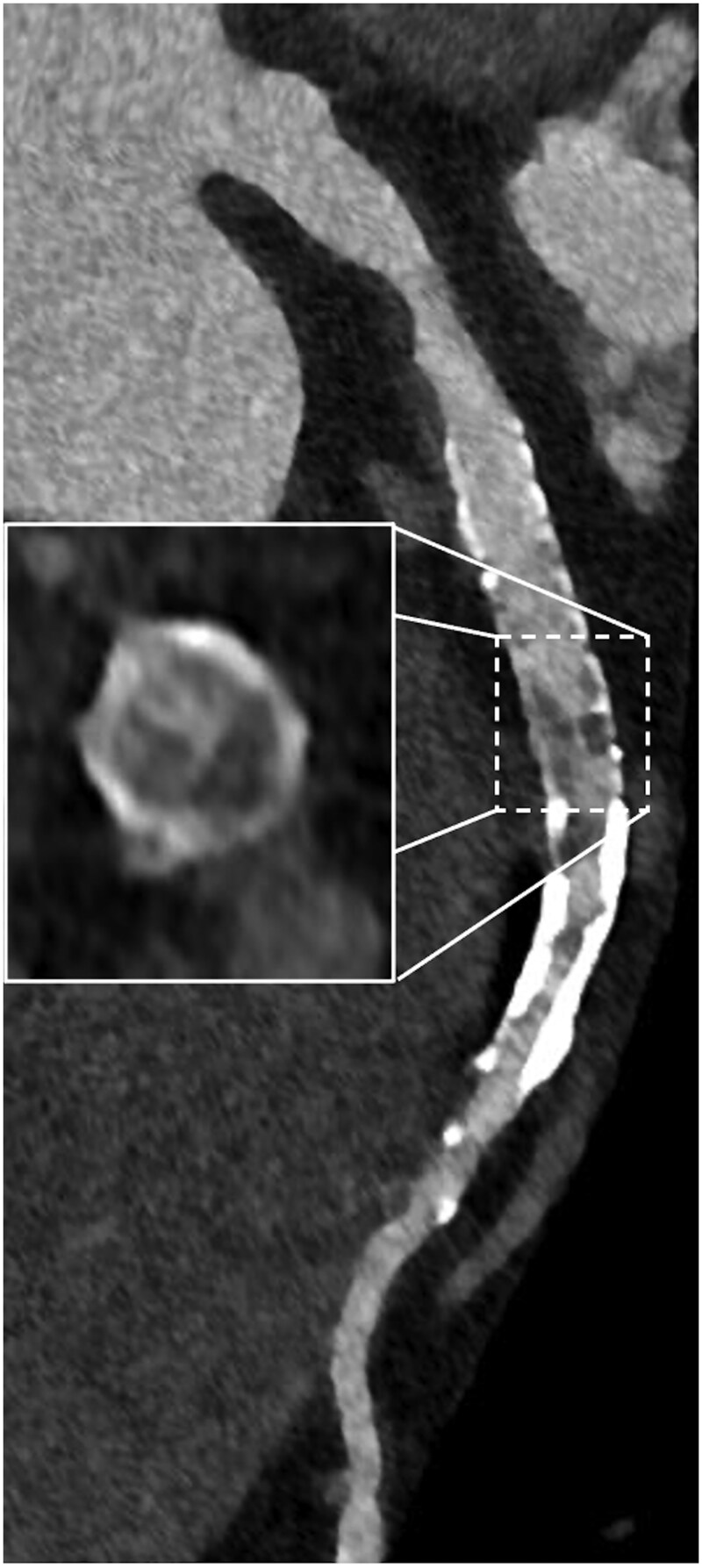
UHR imaging of stents. There is a long stented segment in the proximal and mid left anterior descending artery. In the mid-section of the stent, there is low attenuation neointimal hyperplasia causing severe stenosis. In the distal half of the stent, there is calcification build-up around the stent causing moderate stenosis.

**Figure 9:**
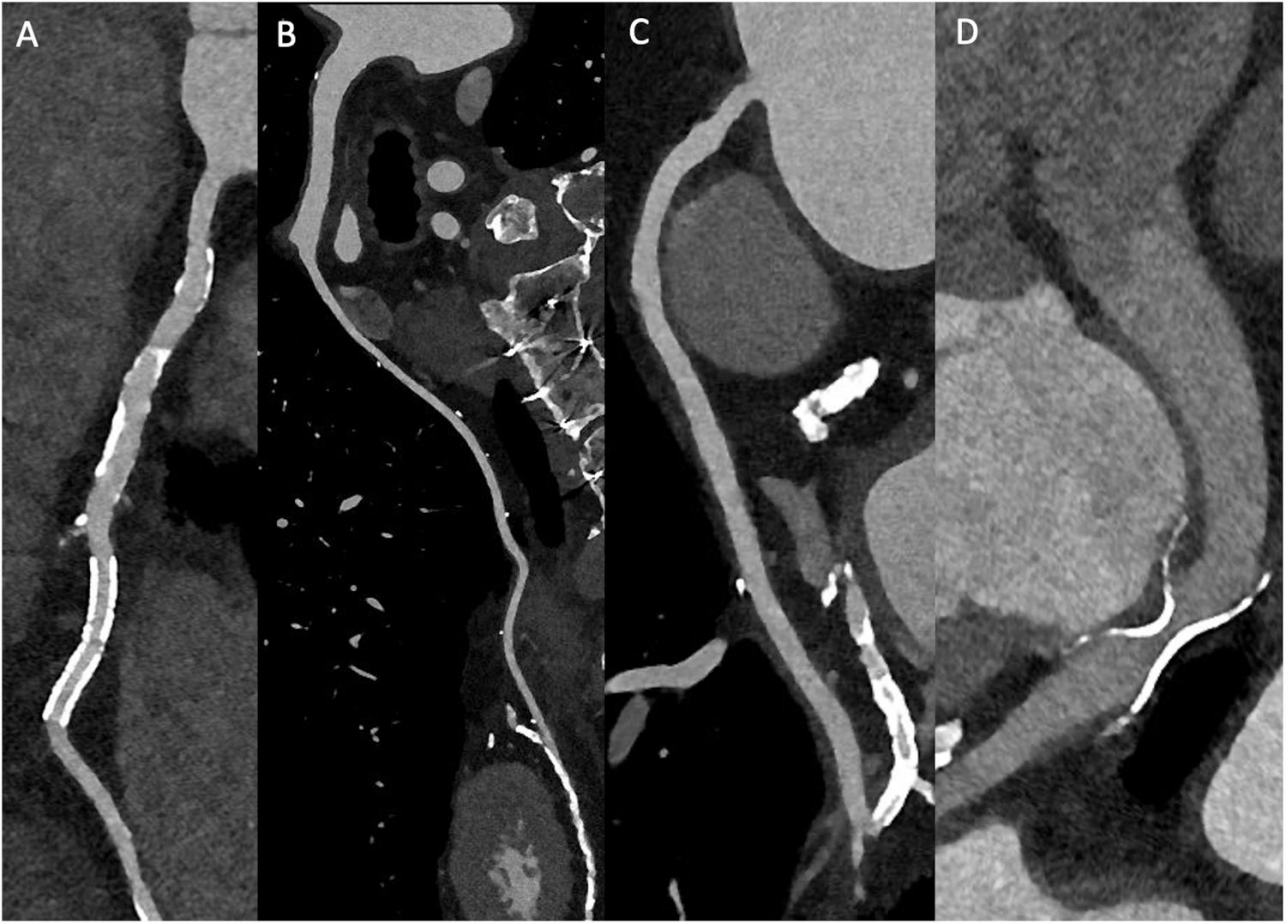
UHR imaging of stent and grafts. UHR protocol with coverage of the entire thorax—patent stent in the distal RCA without severe stenosis (Panel A), the left internal mammary artery (LIMA) graft to the LAD (Panel B), a saphenous vein graft (SVG) to the obtuse marginal branch (Panel C), and a coronary sinus reducer (Panel D), all in one complete acquisition.

For coronary stent evaluation, a UHR scan provides superior spatial resolution and metallic artifact reduction to either FLASH or EID-CT protocols. Figure 8 illustrates a long-stented segment in the proximal and mid left anterior descending artery (LAD). Proximal to the stent there is non-calcified plaque causing mild stenosis. Within the stent, there is low-attenuation neointimal hyperplasia causing severe in-stent restenosis. In the distal half of the stent, there is calcification buildup around the stent causing moderate stenosis. In another patient with coronary artery bypass grafts and a coronary stent (Figure 9A-C), the UHR protocol was used to cover the entire thorax. The UHR images allowed assessment of the patent stent in the distal right coronary artery, the left internal mammary artery graft to the LAD, and the saphenous vein graft to the obtuse marginal branch. This patient also had a coronary sinus reducer device in situ, which required evaluation (Figure 9D). This entire UHR scan was performed using a single phase (75% RR interval) spiral acquisition, 110 mL of contrast, with a resultant radiation dose of DLP 550 mGy.cm (15 mSv).

The UHR protocol should be used with caution in patients with high Body Mass Index (BMI) as the resultant images often have high noise levels. It is preferable to use either a FLEX or spiral acquisition with high IQ level (see below in Arrhythmia protocol).

## Arrhythmia protocol

When the heart rate is 65-70 bpm and regular, a spiral diastolic acquisition 70%-80% of the RR interval provides an adequate phase range for assessment. When the heart rate is >70 bpm and regular, we perform a spiral systolic acquisition 35%-45% RR interval. When the heart rhythm is irregular, we use “padding” or a wider acquisition interval to ensure multiple (particularly end-systolic) phases for assessment. An additional decision is whether to use a FLEX or spiral acquisition mode. The FLEX acquisition is a “step and shoot” approach in which the acquisition is performed in trans-axial blocks of slices. This technique yields lower radiation doses than a spiral acquisition: a mean of 405 mGy.cm (11 mSv) vs 506 mGy.cm (14 mSv) in 50 patients scanned using a FLEX or spiral acquisition, respectively. The FLEX approach is preferred when there are infrequent ventricular ectopic beats, where the acquisition can skip over an occasional ectopic and start in the acquisition in the following RR interval. However, it cannot cope with multiple ventricular ectopic beats. Another limitation of a FLEX acquisition is its tendency to produce step artifacts when a patient struggles with breath-holding. A spiral acquisition can overcome this drawback since the acquisition is continuous, but this does come with an attendant increase in radiation dose.

## Spectral imaging

**Figure 10:**
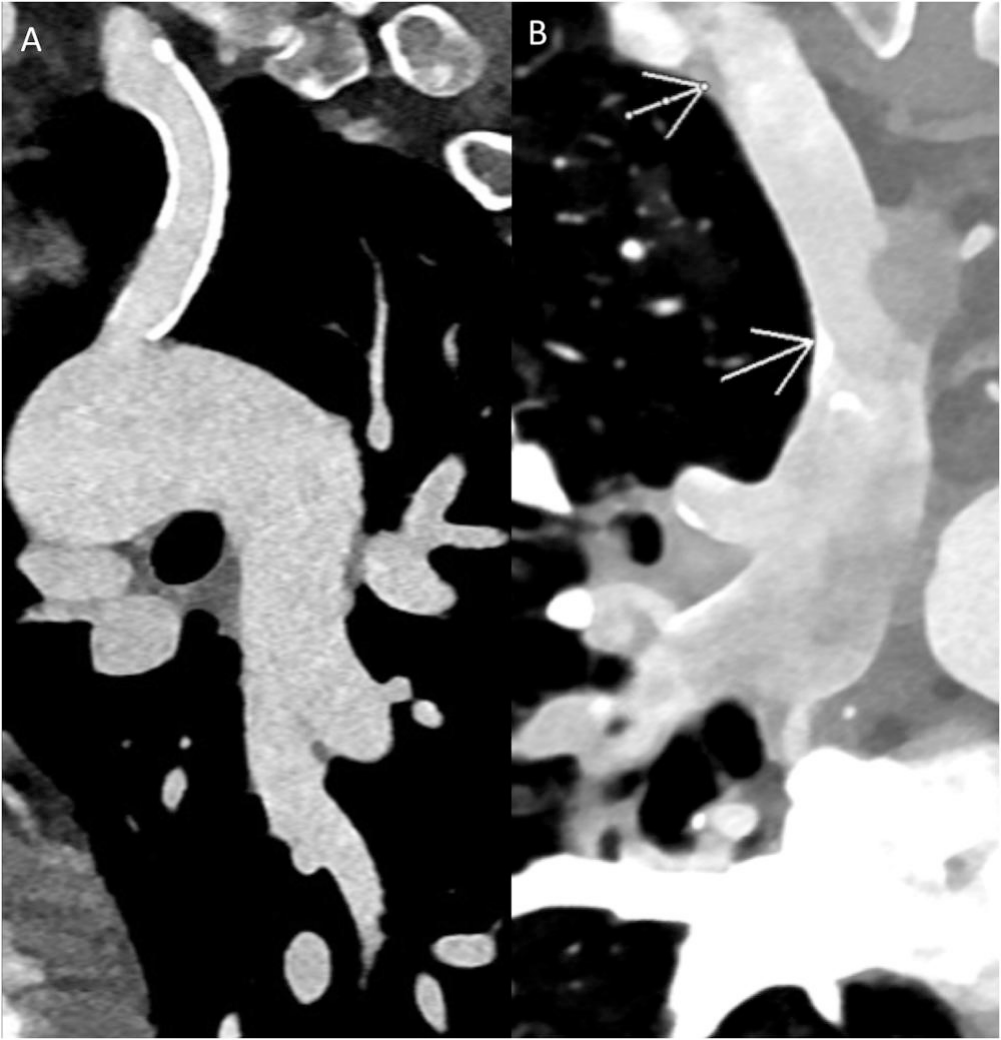
Virtual monoenergetic imaging for conduit assessment in complex congenital heart disease. At 67 keV, the patency of the left-sided Blalock-Taussig (BT) shunt connecting the left subclavian artery to the left pulmonary artery is demonstrated (Panel A). Further scan interpretation at the 50 keV level improves the contrast attenuation and visualization to demonstrate Glenn shunt patency (Panel B) without the need for additional venous phase imaging.

Photon-counting detector CT is capable of spectral data acquisition which enables virtual monoenergetic imaging and material decomposition maps for distinguishing iodine from calcium. These tools can be applied to images acquired through the standard pathway but are not routinely available on UHR reconstructions with 0.2-mm slice thickness. From our experience, virtual monoenergetic imaging has been useful in the following scenarios. Figure 10 illustrates a patient with complex congenital heart disease, where spectral imaging permitted assessment of both arterial and venous conduits using a single acquisition. When the scan was interpreted at 67 keV, we were able to assess the patency of the left-sided Blalock-Taussig (BT) shunt from the left subclavian artery to the left pulmonary artery (Figure 10A). Further scan interpretation at the 50 keV level improves the contrast attenuation and visualization to demonstrate patency of the Glenn shunt connecting the superior vena cava to the right pulmonary artery (Figure 10B). This eliminated the need for additional venous phase acquisition, which had previously been required on the EID-CT.

**Figure 11:**
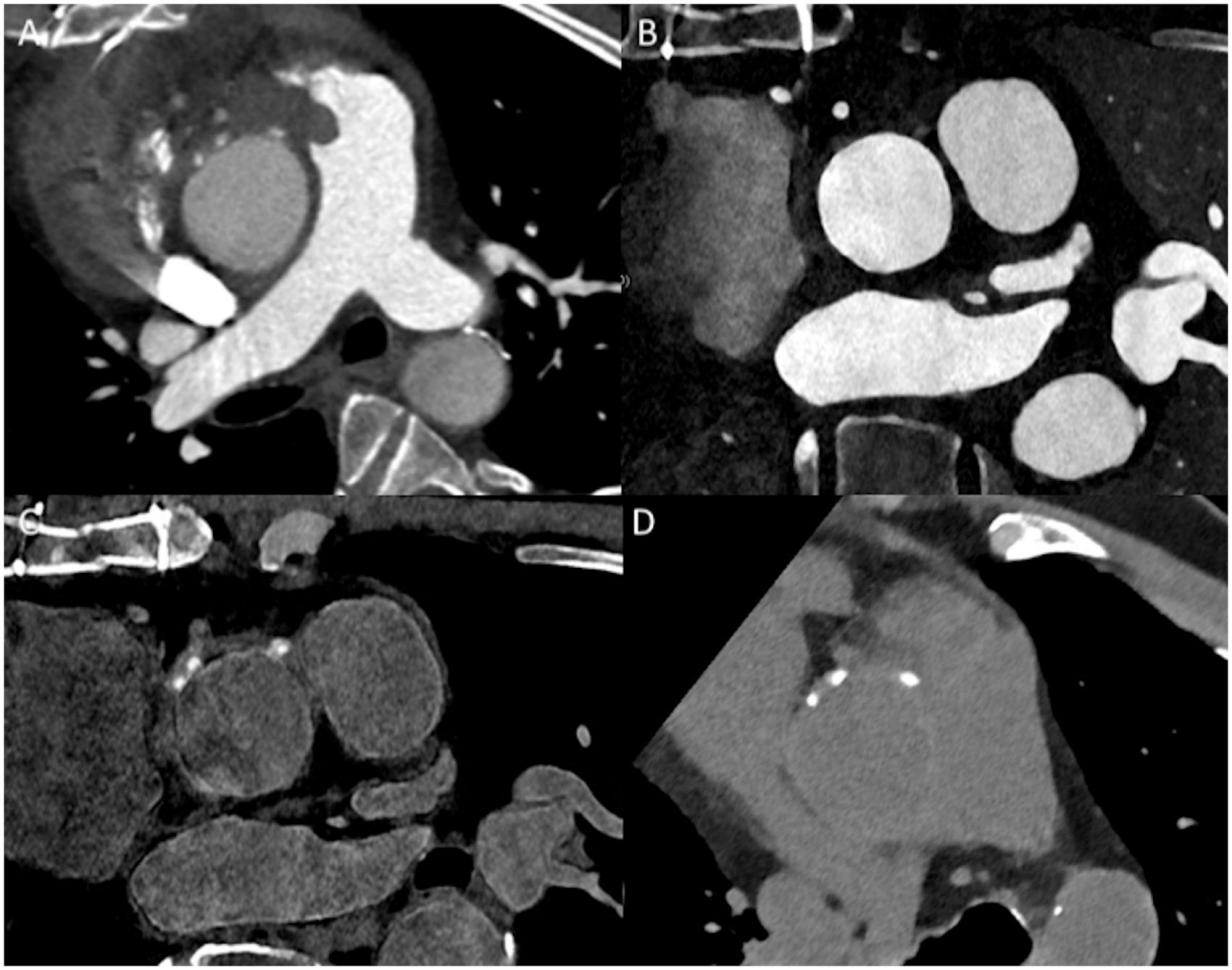
Material decomposition maps in a patient who presented 3 weeks post aortic root abscess debridement patch repair. The abnormality on CTPA (Panel A) was further evaluated on PCD-CT using an iodine only image (Panel B) and a virtual non-contrast image (Panel C) to demonstrate intramural calcification/surgical clips. This was confirmed with a true non-contrast image (Panel D).

Post-acquisition reconstruction with material decomposition can alleviate the need for extra non-contrast images. Figure 11 shows a patient imaged 3 weeks after aortic root abscess debridement and patch repair. A CT pulmonary angiogram (CTPA) revealed a hypodense area at the prior abscess site abutting the pulmonary artery, and within this area, there were presumed high-density enhancement and active leak could not be excluded (Figure 11A). Further assessment with spectral imaging on PCD-CT enabled generation of iodine-only (Figure 11B) and virtual non-contrast (Figure 11C) images, which clarified that the high-density foci correspond to intramural calcification/surgical clips rather than a collection with active contrast leak. A true non-contrast scan confirmed the virtual findings (Figure 11D).

**Figure 12:**
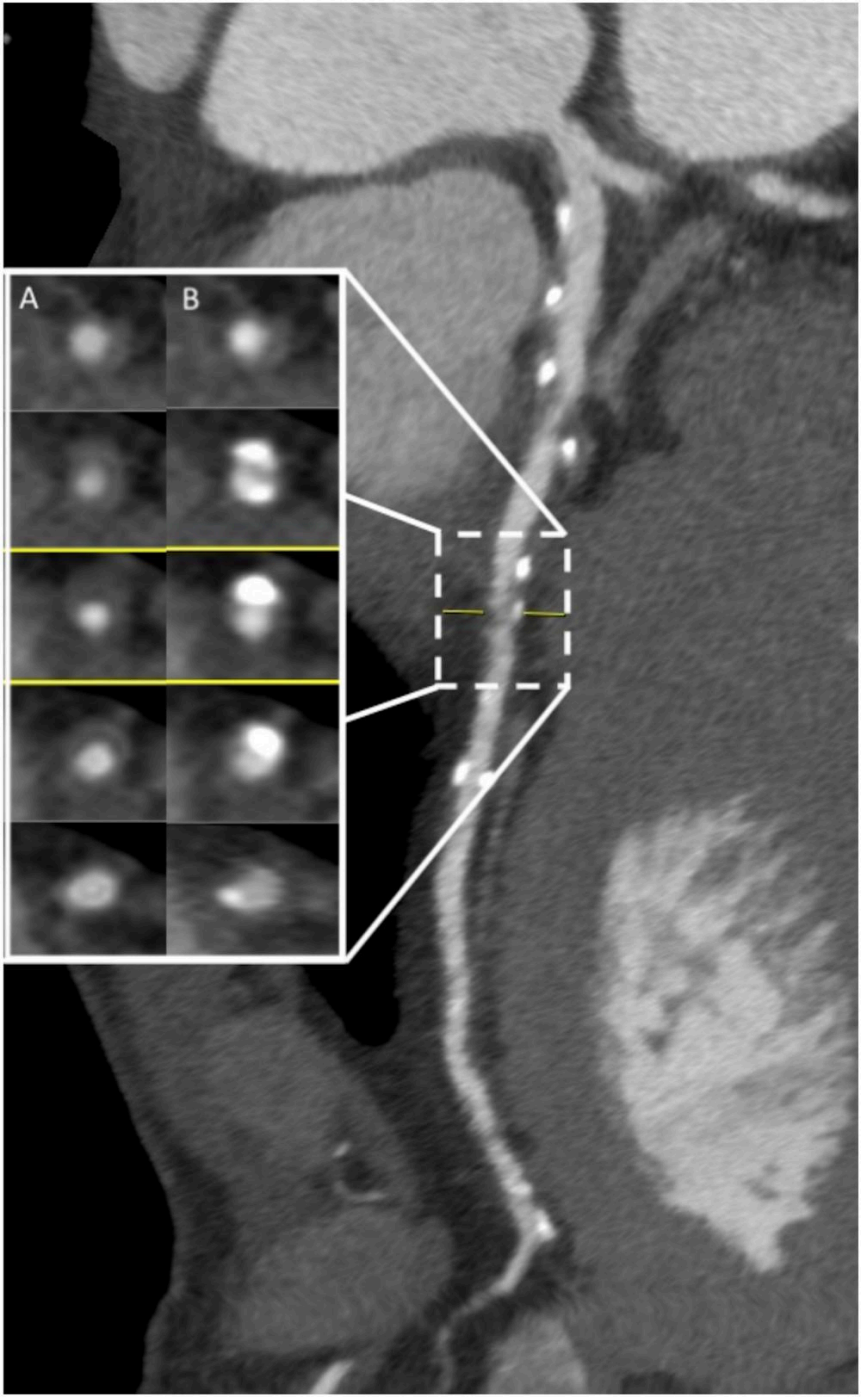
A mixed plaque with improved luminal delineation on cross-sectional imaging after Pure Lumen processing (Panel A) compared with standard reconstruction (Panel B).

**Figure 13:**
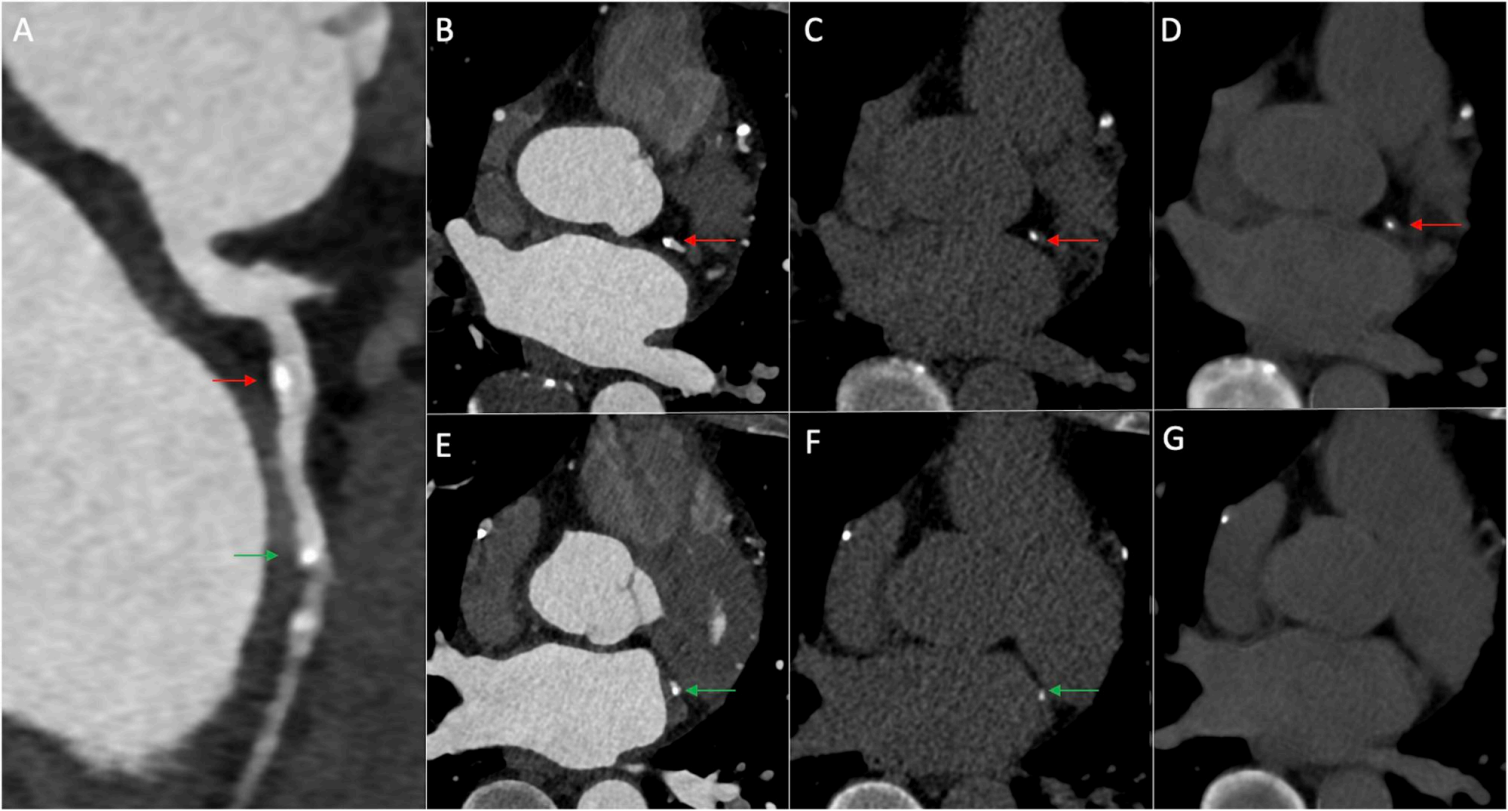
Panel A shows multiplanar reconstruction of the circumflex artery (LCX), and there is a calcified plaque in the proximal LCX (red arrow), and another smaller calcified plaque in the mid-LCX (green arrow). The proximal calcified plaque is visualized on the contrast angiogram (Panel B), detected on both TC (Panel C) and PC (Panel D). The smaller calcified plaque in the mid-LCX is visualized on the contrast angiogram (Panel E), detected in TC (Panel F) but not detected in PC (Panel G).

Post-acquisition reconstruction applications, Pure Lumen and Pure Calcium (PC) can further aid evaluation of complex coronary lesions. The Pure Lumen application subtracts calcium from CCTA data for preferential visualization of the lumen. In Figure 12, a mixed plaque shows improved luminal delineation on cross-sectional imaging after Pure Lumen processing (Panel A) compared with standard multi-planar reconstruction (Panel B). Conversely, the Pure Calcium application removes iodine and could potentially allow calcium scoring without a dedicated non-contrast scan.^16^ However, we have identified discrepancies between Pure Calcium (PC) and True Calcium (TC) measurements at the level of individual coronary vessels (Figure 13), and this feature requires further refinement before routine use.

## Summary

Photon-counting detector CT with its high spatial resolution allowing detailed evaluation of atherosclerotic plaque is pushing the boundaries of coronary imaging. In the clinical setting, it is important to be familiar with the most appropriate scanning protocols and parameters to maximize the clinical impact of PCD-CT.
